# Prevalence of Self-Reported Prediabetes Among Adults Participating in a Community-Based Health Awareness Program, New York State

**Published:** 2009-03-15

**Authors:** Akiko S. Hosler

**Affiliations:** Department of Epidemiology and Biostatistics, University at Albany School of Public Health, and Albany Prevention Research Center

## Abstract

**Introduction:**

The purpose of this study was to assess crude, age-adjusted, and risk-factor–specific prevalences of self-reported prediabetes and to identify factors associated with self-reported prediabetes in an adult population.

**Methods:**

Data were collected through questionnaires completed by a racially diverse sample of diabetes-free adult participants in the statewide community-based wellness and diabetes awareness program in New York State during 2006 (N = 2,572). Prediabetes was determined by the affirmative answer to the question, "Have you ever been told by a doctor that you have prediabetes?"

**Results:**

The overall crude prevalence of self-reported prediabetes was 9.1%, and the age-adjusted prevalence was 7.6%. The age-adjusted prevalence of prediabetes was significantly lower among non-Hispanic blacks (4.2%) and significantly higher among American Indians (22.4%), compared with the prevalence among non-Hispanic whites (7.3%). The prevalence of self-reported prediabetes was uniformly higher among older (aged ≥45 years) adults than younger (aged <45 years) adults, overall and in each racial/ethnic group. In all age and racial/ethnic groups, the prevalence significantly increased with the number of additional risk factors. The best fit multivariate logistic regression model identified that self-reported prediabetes was associated with family history of diabetes (odds ratio [OR], 3.65), body mass index 25.0 kg/m^2^ or higher (OR, 2.79), age 45 years or older (OR, 2.77), and having health insurance (OR, 2.38).

**Conclusion:**

This study found that adults who were at high risk for diabetes and had health insurance were more likely to report having prediabetes. Community-based diabetes prevention needs to consider strategies to increase detection of prediabetes in high-risk uninsured people and to raise general awareness of prediabetes.

## Introduction

Type 2 diabetes, which affects nearly 10% of the US adult population, is a major cause of morbidity, mortality, and decreased quality of life, causing high economic costs (1,2). The prevalence of type 2 diabetes has increased considerably during the past few decades, and the trend is likely to continue through the middle of this century (3,4). An estimated 1 in 3 Americans born in 2000 will develop diabetes in his or her lifetime (5). All people with type 2 diabetes go through a clinical phase called prediabetes (6). Prediabetes is characterized by blood glucose levels that are higher than normal but not in the diabetic range and is defined by having impaired fasting glucose (IFG) or impaired glucose tolerance (IGT) or both (7). IFG is defined by a fasting plasma glucose level of 100 to 125 mg/dL (5.6 to 6.9 mmol/L), and IGT is defined by a 2-hour plasma glucose level of 140 to 199 mg/dL (7.8 to 11.1 mmol/L) after administration of 75 g of oral glucose (7). These ranges of plasma glucose levels signify the threshold at which the risk of type 2 diabetes increases sharply (7). Prediabetes is a serious health condition. People with prediabetes have 10 times the risk of developing type 2 diabetes within 7 years compared with people whose glucose level is in the normal range (8). Prediabetes is also an independent risk factor for cardiovascular disease (9,10).

Scientific evidence suggests that people with prediabetes can delay or reverse the progression to type 2 diabetes. Randomized clinical trials around the world found that interventions with pharmacologic agents (11,12) or lifestyle changes (12-15) can delay or prevent the onset of type 2 diabetes in adults with prediabetes. The Diabetes Prevention Program (DPP), which involved racially and ethnically diverse adults from 27 sites across the United States (16), provides the most relevant evidence for American adults. The DPP demonstrated that in overweight adults with prediabetes, lifestyle modification including modest weight loss, dietary change, and increased physical activity reduced the risk of developing type 2 diabetes by 58%, while drug intervention with metformin reduced the risk by 31% (12). Lifestyle modification was highly effective for all age, sex, and racial/ethnic groups (12).

Prediabetes can only be detected through routine or opportunistic glucose screening. The American Diabetes Association (ADA) recommends periodic glucose screening for people aged 45 years or older, particularly those with a body mass index (BMI) of 25.0 kg/m^2^ or more (17). The ADA also recommends screening for people younger than 45 who have a BMI of 25.0 kg/m^2^ or higher if they have another risk factor for type 2 diabetes, such as family history of diabetes or being a member of high-risk racial/ethnic groups including African American, Hispanic, American Indian, and Asian/Pacific Islander (17). Other medical professional organizations, government agencies, and expert bodies also have age- and risk-based, distinctive recommendations for glucose screening (18,19).

According to the National Health and Nutrition Examination Survey (NHANES), which examines biological samples of respondents, 26% of US adults aged 20 years or older (20) and 7% of US adolescents aged 12 to 19 (21) had prediabetes as measured by IFG. The prevalence of prediabetes increased with age, and significant differences were found by sex and race/ethnicity; men and boys had higher prevalence than did women and girls, and Mexican Americans had higher prevalence than did non-Hispanic whites (20,21). At least 54 million adults (5) and 1.9 million adolescents (21) in the United States are estimated to have prediabetes.

Despite the extent of prediabetes in the US population, scant data are available for the prevalence of self-reported physician-diagnosed prediabetes. Since 2004, the Behavioral Risk Factor Surveillance System (BRFSS) telephone survey has been coding "prediabetes or borderline diabetes" as an unsolicited answer option to the question for diagnosed diabetes (22). The 2006 data indicate that the nationwide prevalence of self-reported prediabetes or borderline diabetes in adults aged 18 years or older was 1.0% (23). The BRFSS questionnaire, however, does not independently and specifically ask respondents if they have been diagnosed with prediabetes.

This study assessed the prevalences of self-reported prediabetes in diabetes-free adults by using a structured questionnaire. The study was conducted to provide crude and age-adjusted prevalences of self-reported prediabetes for the overall population and population strata. To examine relationships between self-reported prediabetes and its related risk factors, both descriptive and multivariate logistic regression analyses were conducted. This study aims to provide baseline information on the extent of self-reported prediabetes in an adult population and to facilitate the formulation of community-based public health strategies to increase awareness and detection of prediabetes, as part of the national effort to translate the DPP results into practice.

## Methods

Data for this study were collected from diabetes-free adults aged 18 years or older who participated in a community-based wellness and diabetes awareness program in New York State sponsored by the state health department. A total of 15 regional core organizations and 146 partner organizations took part in the program to assure geographic coverage of all 62 counties in the state. Participants were volunteers who responded to program announcements made by local partner organizations through various outreach channels. The only criteria for participation were being age 18 years or older and having no prior diagnosis of diabetes. From January 1 through December 31, 2006, all participants were asked to complete a questionnaire at registration. The New York State Department of Health Institutional Review Board reviewed and approved this study. No identifiable personal information was requested to ensure complete anonymity. The questionnaire was a single-page form, written at a sixth-grade reading level and made available in both English and Spanish.

The questions included were age, sex, race/ethnicity, physician-diagnosed prediabetes, gestational diabetes, BMI, family history of diabetes, tobacco product use, and health insurance coverage. These questions were selected on the basis of established risks for prediabetes and type 2 diabetes, availability of tested and widely used survey questionnaires, and appropriateness for the diverse population. Diagnosed prediabetes was assessed through the question, "Have you ever been told by a doctor that you have prediabetes?" The technical terms IFG and IGT or the colloquial "borderline diabetes" were not used because prediabetes is the preferred term for provider-patient communication and health education (24,25). Gestational diabetes was assessed if the respondent had diabetes only during pregnancy. For BMI information, the respondent was instructed to select 1 of the 4 letter-coded sections (A to D) in the height and weight chart where his or her height and weight fell. The sections were designed to correspond to underweight (BMI <18.5 kg/m^2^), normal weight (BMI, 18.5-24.9 kg/m^2^), overweight (BMI, 25.0-29.9 kg/m^2^), and obese (BMI ≥30.0 kg/m^2^). Family history of diabetes specifically referred to diagnosed diabetes in mother, father, sister, or brother. The tobacco-use question was phrased as, "Do you use any tobacco products regularly?" The health insurance coverage question was, "Do you have a health insurance plan that covers most of your health care costs?"

A total of 2,572 people completed the entire questionnaire. I obtained the overall and stratum-specific crude prevalence of prediabetes. For the purpose of comparison, I age-adjusted the overall and stratum-specific prevalence to the 2000 New York State Census population by using the direct method, with age categories of 44 years or younger, 45 to 64 years, and 65 years or older. The race/ethnicity prevalence analysis was limited to 4 groups (non-Hispanic white, non-Hispanic black, Hispanic, and American Indian) that had sufficient sample size (N ≥ 50) for stable prevalence estimation and age-adjustment calculation. I calculated the 95% confidence intervals by using the Fisher exact method.

In the next analysis, I grouped the study population into age- and race/ethnicity-specific groups on the basis of the known risk for prediabetes. The population was first separated by age (aged ≥45 years and <45 years), then divided into non-Hispanic white and minorities (Hispanics and/or nonwhites). These categories were further stratified into mutually exclusive and exhaustive combinations of 2 additional risk factors: BMI 25.0 kg/m^2^ or higher and family history of diabetes. I obtained the prevalence and 95% confidence intervals of self-reported prediabetes for all strata. I calculated the χ^2^ trend statistic for prevalence by combined risk factors (0, 1, or 2) for each demographic cluster.

Finally, I constructed logistic regression models to identify factors associated with self-reported prediabetes. First, all independent variables (age, sex, race/ethnicity, BMI, family history, gestational diabetes, tobacco use, and health insurance coverage) were entered in the model, and then the backward stepwise selection method was used to remove nonsignificant variables. For the purpose of demographic control, I forced age, sex, and race/ethnicity variables to remain in the initial model. I also tested 2 interaction terms: insurance status by race and BMI by race. I continued the variable removal process to obtain the best fit model, this time by examining the Hosmer-Lemeshow goodness-of-fit statistic. I present the initial and best fit models with the odds ratios, 95% confidence intervals (CIs) of the odds ratios, and their significance.

## Results

The age distribution of the sample was 32.7% for the group aged 18 to 44 years, 37.7% for 45 to 64 years, and 29.5% for 65 years and older. Approximately 41% of the sample were racial/ethnic minorities, including non-Hispanic black (24.1%), Hispanic (9.1%), American Indian (3.3%), Asian and Pacific Islander (1.2%), and mixed or other races (2.9%). In New York State, approximately 35% of the adult population (aged ≥18 years) are racial/ethnic minorities.

The overall crude prevalence of self-reported prediabetes in this sample was 9.1% (95% CI, 8.0-10.2). The prevalence increased with age (Table 1); the age-adjusted overall prevalence was 7.6% (95% CI, 6.6-8.7). After age adjustment, American Indians had higher rates of prediabetes than did non-Hispanic whites, and non-Hispanic blacks had lower rates of prediabetes than did non-Hispanic whites. Prediabetes was significantly more prevalent among adults with BMI of 25 kg/m^2^ or higher. Prevalence was also higher in adults who had a family history of diabetes and in adults with health insurance. No significant difference between men and women was observed (Table 1).

In the analysis of risk-factor–specific prevalence of self-reported prediabetes, several general trends were observed. The prevalence was higher in the older group (aged ≥45 years) compared with the younger group (aged <45 years) (11.3% vs 4.4%) for the study population overall. Higher prevalence in the older group compared with the younger group was also observed among non-Hispanic whites (12.4% vs 3.1%) and among minorities (9.4% vs 5.6%). Within each age and racial/ethnic subgroup, prevalence increased significantly with each additional risk factor. The trend was exponential rather than linear, as the increase of prevalence accelerated from 1 risk to 2 risks (Table 2).

In the logistic regression analysis, the initial model with demographic control variables found that people who had a family history of diabetes, BMI ≥25.0 kg/m^2^, age 45 years or older, and health care coverage were 2.3 to 3.6 times more likely to have self-reported prediabetes than were those who did not have those properties. In terms of race and ethnicity, American Indians were approximately 3.6 times more likely to report prediabetes than was any other race category, while for non-Hispanic blacks the probability of reporting prediabetes was almost half that of other racial groups. This model, however, had a poor overall fit, indicated by the Hosmer and Lemeshaw goodness-of-fit test statistic (χ^2^ = 10.9, *df* = 8, *P* = .2). The interaction terms did not contribute to any significant improvement of the model. The best fit model (χ^2^ = 2.18, *df* = 7, *P* = .94) removed the race and sex variables (Table 3).

## Discussion

The findings of this study showed a significant association between health insurance coverage and diagnosed prediabetes. Access to affordable health care appears to be a prerequisite for being diagnosed with prediabetes. Despite differences in the eligibility criteria for screening, the ADA, the US Preventive Services Task Force, and other influential US medical organizations and government agencies recommend practice-based, in-clinic glucose screening (18,19,26). Mass community-based glucose screening is discouraged because the number of diabetes and prediabetes cases diagnosed is small (27) and its standardized methods and cost-effectiveness have not been established (18). People without health insurance are less likely to have routine checkups, and their lower use of nonemergency clinical facilities may reduce their chances of receiving opportunistic glucose screening (28). Access to affordable health care is critical for both diagnosis of prediabetes and subsequent lifestyle and pharmacologic intervention to prevent progression to type 2 diabetes.

American Indians' high prevalence of self-reported prediabetes was notable. American Indians have been recognized for their biological susceptibility to abnormalities in glucose metabolism (29). More rigorous glucose screening starting at a younger age and increased awareness of prediabetes for this population may have contributed to the high prevalence. The Indian Health Service recommends that American Indian adults aged 18 years or older should be tested for prediabetes annually if they have 1 or more diabetes risk factors, and if no risk factors exist, testing should begin at age 35 and be repeated a minimum of every 3 years (30). In fact, the prevalence of self-reported prediabetes among American Indian adults younger than 45 years was 21%, which was 6 to 10 times higher than that of other racial/ethnic groups in the same age range (data not shown).

The lower prevalence of self-reported prediabetes among non-Hispanic blacks compared with that among non-Hispanic whites is consistent with previous findings. Studies using NHANES data have reported uniformly lower prevalence of IFG, IGT, and prediabetes among non-Hispanic black adults and adolescents compared with that among non-Hispanic whites (20,21,31). One possible hypothesis is that type 2 diabetes progresses more aggressively in blacks, resulting in a relatively short prediabetic phase. NHANES studies reported a higher prevalence of prediabetes among Mexican Americans, but in this study, Hispanics had a slightly lower prevalence than did non-Hispanic whites. New York State Hispanics mostly originate from the Caribbean and Central America (32), so a direct comparison with Mexican Americans may not be relevant. Linguistic or cultural barriers may limit the rate of glucose screening among this group, resulting in a lower prevalence of diagnosed prediabetes. The association of race/ethnicity with diagnosed prediabetes was only marginally significant. Further research is needed to understand the role of race/ethnicity in the diagnosis of prediabetes.

There are limitations in this study. The precision of estimate was compromised by the small sample size. Because the study was based on self-report, biases may have resulted from respondents' recall. Some respondents probably have had blood glucose in the prediabetic range but were not informed of their prediabetes status by their physicians. Most laboratory reports use the terms IFG and IGT to indicate prediabetes, so patients need to be educated by their physicians about what these terms mean. Some physicians probably choose to use "borderline diabetes" or "a touch of sugar" to inform patients about the glucose abnormality.

In the United Kingdom, physicians who lack knowledge and skills to promote lifestyle changes, have large workloads, and believe health promotion is not a role of physicians are less likely to discuss prediabetes with their patients (33-35). Little is known about how US physicians inform and educate their patients about prediabetes. This study did not collect information regarding specialty and practice type of the physicians that respondents were seeing.

Another source of bias is the healthy volunteer effect. This sample, however, was older and included more people who were obese or were members of high-risk minority populations than the state's adult population without diagnosed diabetes, according to 2006 BRFSS data. Awareness of prediabetes and participation in glucose screening among the sample might be higher than that of the state's general adult population.

This study provides a knowledge base of the extent of self-reported prediabetes and its associations with selected risk factors in racially diverse adults in a community setting. This type of information is highly desirable for the formulation of community-based strategies to detect prediabetes and prevent type 2 diabetes. This study demonstrates that people who lack health insurance are less likely to report prediabetes than are those who have insurance. As efforts to translate the findings of the DPP into community settings continues, there is an urgent need to increase general awareness of prediabetes and allocate public health resources to systematically identify and follow uninsured or underinsured high-risk people with prediabetes. In addition, more research is needed to understand the roles of race and ethnicity and physician-patient communication in the diagnosis of prediabetes.

## Figures and Tables

**Table 1:** Crude and Age-Adjusted Prevalence (%) of Self-Reported Physician-Diagnosed Prediabetes in Adults Participating in Diabetes Awareness Program, New York State, 2006

Category	No.	Crude		Age-Adjusted	

		% (95% CI)	*P* value	% (95% CI)	*P* value
**Age, y**					
<45	842	4.4 (3.1-6.0)	Ref	—	—
45-64	970	10.8 (8.9-13.0)	<.001	—	—
>65	760	12.0 (9.8-14.5)	<.001	—	—
**Sex**					
Male	528	8.1 (6.1-10.5)	.35	6.5 (4.7-8.8)	.25
Female	1,944	9.4 (8.1-10.7)		7.9 (6.8-9.2)	
**Race/ethnicity^a^**					
Non-Hispanic white	1,531	9.9 (8.4-11.5)	Ref	7.3 (6.0-8.7)	Ref
Non-Hispanic black	619	4.7 (3.2-6.7)	<.001	4.2 (2.8-6.1)	.008
Hispanic	235	6.4 (3.6-10.3)	.09	6.0 (3.3-9.8)	.45
American Indian	85	23.5 (15.0-4.0)	<.001	22.4 (14.0-2.7)	<.001
**Body mass index, kg/m^2^**					
<25.0	974	4.2 (3.0-5.7)	Ref	3.5 (2.4-4.8)	Ref
25.0-29.9	825	8.1 (6.3-10.2)	<.001	13.5 (11.3-6.1)	<.001
≥30.0	773	16.2 (13.6-9.0)	<.001	14.1 (11.7-6.8)	<.001
**Family history of diabetes**					
Yes	1,069	15.4 (13.3-7.7)	<.001	13.5 (11.5-5.7)	<.001
No	1,503	4.5 (3.5-5.7)		3.5 (2.7-4.6)	
**Gestational diabetes**					
Yes	65	7.7 (2.5-17.0)	.64	7.7 (2.5-17.0)	.92
No	1,879	9.4 (8.1-10.8)		8.0 (6.8-9.4)	
**Regular tobacco use**					
Yes	357	7.8 (5.3-11.1)	.39	8.6 (6.0-12.1)	.45
No	2,215	9.3 (8.1-10.5)		7.5 (6.5-8.7)	
**Health insurance**					
Yes	2,232	9.8 (8.6-11.1)	.001	8.2 (7.1-9.4)	.005
No	340	4.1 (2.3-6.8)		3.8 (2.1-6.5)	
All adults	2,572	9.1 (8.0-10.2)	—	7.6 (6.6-8.7)	—

Abbreviations: CI, confidence interval; Ref, reference group.

a Sample size for Asian and Pacific Islanders, mixed races, and other races was too small (<50) to provide stable prevalence estimates and age-adjustment calculation

**Table 2:** Prevalence of Self-Reported Physician-Diagnosed Prediabetes in Adults Participating in Diabetes Awareness Program, by Age, Race/Ethnicity, and Combination of Risk Factors, New York State, 2006

Combination of Risk Factors	All Races		Non-Hispanic Whites		Racial/Ethnic Minorities^a^	

	No.	% (95% CI)	No.	% (95% CI)	No.	% (95% CI)
Age ≥45 y	1,730	11.3 (9.9-2.9)	1,114	12.4 (10.5-4.5)	616	9.4 (7.2-12.0)
No added risks	426	3.5 (2.0-5.7)	308	4.2 (2.3-7.1)	118	1.7 (0.2-6.0)
BMI ≥25.0 kg/m^2^	588	8.0 (5.9-10.5)	379	8.7 (6.1-12.0)	209	6.7 (3.7-11.0)
Family history of diabetes	220	9.6 (6.0-14.2)	141	10.6 (6.1-16.9)	79	7.6 (2.8-15.8)
BMI ≥25 kg/m^2^ and family history of diabetes	496	22.8 (19.2 -26.7)	286	26.9 (21.9-2.5)	210	17.1 (12.3-22.9)
*P* value^b^	<.001		<.001		<.001	
Age <45 y	842	4.4 (3.1-6.0)	417	3.1 (1.7-5.3)	425	5.6 (3.7-8.3)
No added risks	207	0 (0.0-1.8)	130	0 (0-2.8)	77	0 (0-4.7)
BMI ≥25 kg/m^2^	282	2.1 (0.8-4.6)	135	3.0 (0.8-7.4)	147	1.4 (0.2-4.8)
Family history of diabetes	121	4.1 (1.4-9.4)	59	5.1 (1.1-14.1)	62	3.2 (0.4-11.2)
BMI ≥25 kg/m^2^ and family history of diabetes	232	11.2 (7.5-16.0)	93	6.5 (2.4-13.5)	139	14.4 (9.0-21.3)
*P* value^b^	<.001		.004		<.001	

Abbreviations: CI, confidence interval; BMI, body mass index.

a Includes nonwhite races and Hispanics.

b *P* values were determined by χ2 test for linear trend.

**Table 3:** Logistic Regression Models for Factors Associated With Self-Reported Physician-Diagnosed Prediabetes in Adults Participating in Diabetes Awareness Program, New York State, 2006

Variable	Model With Demographic Controls^a^		Best Fit Model^b^	

	OR (95% CI)	*P* value	OR (95% CI)	*P* value
**Family history of diabetes**				
Yes	3.56 (2.61-4.85)	<.001	3.65 (2.70-4.92)	<.001
No	1 [Reference]		1 [Reference]	
**Body mass index, kg/m^2^**				
≥25	2.78 (1.94-3.97)	<.001	2.79 (1.96-3.97)	<.001
<25	1 [Reference]		1 [Reference]	
Age, y				
≥45	3.14 (2.12-4.64)	<.001	2.77 (1.91-4.00)	< .001
<45	1 [Reference]		1 [Reference]	
**Health care coverage**				
Yes	2.32 (1.28-4.22)	.006	2.38 (1.35-4.18)	.003
No	1 [Reference]		1 [Reference]	
**Race^c^**				
Non-Hispanic white	0.96 (0.96-1.77)	.90	—	
Non-Hispanic black	0.44 (0.20-0.99)	.048		
Hispanic	0.55 (0.22-1.34)	.19		
American Indian	3.63 (1.63-9.09)	.001		
All other	1 [Reference]	—		
**Sex^c^**				
Male	1.22 (0.86-1.73)	.66	—	
Female	1 [Reference]			

a The Hosmer-Lemeshow goodness-of-fit test was used to assess overall fit (χ28 = 10.9, *P* = .21).

b The Hosmer-Lemeshow goodness-of-fit test was used to assess overall fit (χ27 = 2.2, *P* = .95).

c Race and sex were removed in the best fit model through the backward stepwise selection method. These variables were found to be nonsignificant based on the probability of a likeihood-ratio statistic.
